# Adhesion Ileus after Fecal Microbiota Transplantation in Long-Standing Radiation Colitis

**DOI:** 10.1155/2019/2543808

**Published:** 2019-01-02

**Authors:** Igor Alexander Harsch, Peter Christopher Konturek

**Affiliations:** ^1^Department of Internal Medicine II, Division of Endocrinology and Metabolism, Rainweg 68, 07318 Saalfeld/Saale, Germany; ^2^Department of Internal Medicine II, Division of Gastroenterology, Rainweg 68, 07318 Saalfeld/Saale, Germany

## Abstract

Fecal microbiota transplantation (FMT) is a novel strategy for the therapy of dysbiosis-associated disorders via modulation of the gut microbiota. Intestinal dysbiosis is associated not only with digestive disorders, but also with a variety of extra-digestive disorders. A worldwide increasing number of FMT can be expected in the future as well as an increase in adverse events. We describe the case of a patient with chronic radiation colitis that developed adhesion ileus 2 days after FMT. Since these problems never occured before and the short time interval favours a causality, we speculate about FMT-induced alterations in gut motility causing a “trapping” of the small intestine in an adhesion and other mechanisms beyond “pure” coincidence.

## 1. Introduction

Fecal microbiota transplantation (FMT) is a promising novel approach in several gastroenterological disorders. FMT has shown promising results in irritable bowel disease [1] in inflammatory bowel disease [2] and is yet an accepted therapeutic option in recurrent clostridium difficile colitis [3, 4]. There are also reports of this approach successful in rarer conditions such as chronic intestinal pseudoobstruction [5]. However, in novel medical approaches, there is a tendency to underreport adverse events. One example is bariatric surgery. In the Cochrane Database of Systematic Reviews J.L.Colquitt et al. [6] state that “across all studies adverse event rates and reoperation rates were generally poorly reported.” The aspect of potential underreporting of adverse events concerning FMT had also been addressed by Wang et al. [7] in their systematic review about adverse events of FMT. The authors mention two randomized controlled trials of FMT in Clostridium difficile colitis [8] and in ulcerative colitis [9] with a high incidence of adverse events in this study setting and regard this suggestive of underreporting in previous studies with another study design.

The healthcare community is assessing FMT with high interest and the patients are becoming more aware of the procedure. Thus, a growing number of FMT can be expected in the future. With an increasing number of a new medical therapy or medication, unexpected side effects may emerge as can be seen in proton pump inhibitors [10]. Although causality is highly debatable, this is why we chose to report a case of adhesion ileus 2 days after FMT.

## 2. Case Presentation

A 56-year-old Caucasian female patient underwent Wertheim-Meigs radical hysterectomy as surgical treatment of cervical carcinoma in 1986. Furthermore, radiotherapy of 30 x 8 GY was performed. Her present BMI was 22 kg/m^2^. She underwent subtotal thyroidectomy because of a cold knot in 2000. Her main medical problem was diarrhea. The patient has been suffering from constant diarrhea for 17 years (stool frequency between 9 and 20 times a day). As part of the diagnostics of the diarrhea H_2_-breath tests with lactose, fructose and sorbitol were performed. She was diagnosed with a lactose and fructose malabsorption. Furthermore, a* Helicobacter pylori* eradication is worth mentioning (2013). Several rectoscopies and colonoscopies (2008, 2013, 2014, and 2016) revealed a radiotherapy-induced stenosis in the area of the sigmoid colon. There were never histologic aspects of inflammatory bowel disease. A computed tomography of the abdomen and pelvis revealed a long-range concentric thickening of the rectal wall with blurred confinement and fluid imbibition of the perirectal fatty tissue (2013). These endoscopic and radiologic findings in combination with the clinical picture confirmed the diagnosis of chronic radiation colitis.

Several conservative therapies were performed, including various probiotics such as* E. coli* strain Nissle 1917, Bifidobacteria (*B. bifidum* MIMBb75), loperamide, metoclopramid, mesalazine, intestinal tea, psyllium, rice cures, healing earth, etc. None of these therapeutic approaches led to a significant and sustained improvement of her symptoms.

Due to these complaints, the quality of life of the patient was extremely reduced, the social contacts suffered from it, and the patient could hardly leave home due to the diarrhea. Therefore, she asked to have carried out a fecal microbiota therapy in order to improve the intestinal dysbiosis and thus also to improve the symptoms.

On June 27, 2018, after giving informed consent to this individual therapy trial, and after 5 days of pretreatment with rifaximin, the patient had FMT from an unrelated donor with negative serum tests for hepatitis B and C, HIV, CMV, EBV,* Treponema pallidum*, and negative stool cultures for costridium difficile toxin, parasites, and worm eggs, as well as noro- and rotaviruses. The colonoscopy was performed until the terminal ileum. Between 20 and 40 cm ab ano, a mucosal atrophy and narrowing of the intestinal lumen as a result of radiation colitis was visible. FMT was done with 500 ml stool graft in terms of ileum, coecum, and colon ascendens. After the procedure, she received 2 x 2 mg loperamide and 3 x 2 mg the following day and was discharged without any symptoms. On June 28, 2018, she had stool one time and, on the following day, she developed an increasing nausea and a sense of increasing meteorism without defecation and winds.

On June 30, 2018, the patient was sent to the emergency room due to these symptoms. She had no fever and had no colics.

**Figure 1:**
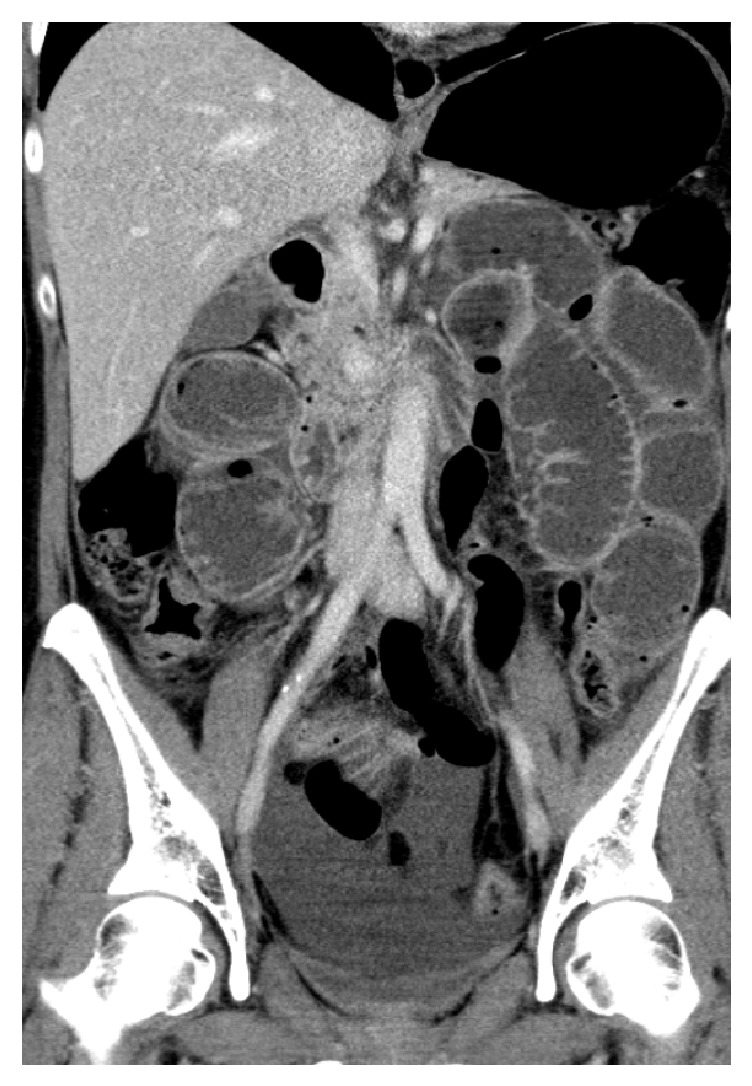
CT of the abdomen: picture of a mechanical small bowel ileus. The colon is not involved. Thus, the caliber jump is most likely in the preterminal ileum, but this cannot be demonstrated on the CT pictures.

In a CT scan, a complete small intestinal ileus could be detected; the colon was not involved (see Figure 1). Conservative therapeutic attempts were unable to improve the result. That said, the indication for surgery on the following day was made.

A median upper and lower abdominal laparotomy and opening of the abdomen were performed. It came to the protrusion of intestinal loops from the abdominal wall, which were distended. In the distal part of the jejunum there was a strangulation of the intestine with an adhesion.

The adhesion was released and cut. This reversed the strangulation of the bowel. A resection of the small intestine was not required. After surgery, the started diet was well tolerated by the patient. The wounds healed* per primam. *After discharge from the department of surgery, the gastrointestinal problems had improved for 3-4 weeks, but did then revert to the state before FMT.

## 3. Discussion

The management of chronic radiation colitis is still a major challenge due to the progressive evolution of the disorder, including aspects such as the development of fibrosis, endarteritis, edema, fragility, partial obstruction, perforation, and the possible development of cancer [11]. Diarrhea, with or without abdominal cramps, is the most common symptom of chronic radiation colitis [11]. Several therapeutical approaches are available or under development, but a significant improvement of the patients clinical symptoms is not always acheivable yet.

A rational to include FMT into the possible therapeutic strategies stems from animal studies.

Garassy-Vainberg et al. [12] could demonstrate that rectal radiation induces dysbiosis, which transmits radiation and inflammatory susceptibility and provide evidence that microbial-induced radiation tissue damage is at least in part mediated by IL-1*β* in a mouse model of radiation colitis. They speculated that the host might take profit via modifications of the microbiome and that its manipulation may potentially allow novel interventional approaches. Protective effects of FMT against radiation-induced toxicity have recently been demonstrated in a mouse model by Cui et al. [13].

From the logic of our medical understanding it is problematic to speculate about an interaction between FMT and a mechanical small bowel ileus. On the other hand, the rapid development of the problem after FMT in a patient that had never had this medical problem before raises doubts, especially in a setting, where our understanding of the interactions between gut microbiota and the host's organs is still fragmentary. This is why we dare to speculate about possible causal interactions and their pros and cons.

(1) Causality due to unknown effects of the newly transplanted bacterial species on gut motility via yet unknown mechanisms. Altered, increased small gut motility may lead to a “trapping” of a gut segment in a preexisting adhesion

Pro: An important impact of gut microbiota on the release of centrally active hormones and neurotransmitters via the production of short-chain fatty acids is known [13]. As for hormones released via SCFAs produced by gut microbiota, gut motility slows after the SCFA-mediated release of Glucagon-like peptide 1 (GLP-1) [14]. Neurotransmitters released via action of gut microbiota do also interact with the autonomic nervous system [15]. In a very recent study, Bhattarai et al. [16] demonstrated in an animal model that the contrary concerning gut motility is also conceivable: germ-free (GF) mice colonized by* Bacteroides thetaiotaomicron* engineered to produce tryptamine exhibit an accelerated gastrointestinal transit. Tryptamine is a tryptophan-derived monoamine produced by gut bacteria and abundant in human and rodent feces. The authors could demonstrate that the biological effects of tryptamine are mediated through the 5-HT_4_ receptor, a G-protein-coupled receptor uniquely expressed in the colonic epithelium.

Cons are as follows: there was no no established pathophysiological concept for increased gut motility in humans yet. Unfortunately, there were no stool-samples available in the postoperative interval to verify a possible change in the composition of the microbiota.

*(2) An Effect of Loperamide Therapy*. Pros are as follows: Loperamide exerts its antidiarrheal effect by a change in the motor function of the intestine, which results in an increased capacitance of the gut and a delay in the passage of fluid through the intestine. Overdose may result in an ileus.

Cons are as follows: the maximum recommended dose of loperamide in acute diarrhea is 16 mg/die. 10 mg in 2 days, as applied in our very case, is not a high dose. Furthermore an ileus problem should have been of paralytic not of mechanical nature. The patient did also have episodes of loperamide therapy (2-3 capsules daily) in the past without any problems.

*(3) Effect of the Procedure Itself*. Pros are as follows: the patient had several previous endoscopic procedures. Perhaps the fluid injection or some other aspect of the procedure (e.g., gas production after the application of the FMT preparation due to unknown reasons) was different enough in this case to cause a mechanical change in the intestinal structure.

Cons are as follows: Since 2014, about 100 FMTs were performed in our clinic in a standardized way; there was no different proceeding in the case reported here.

*(4) “Pure” Coincidence*. Pros were as follows: there was no established pathophysiological concept between effects of the gut microbiota and ileus or previous reports about such phenomena.

Cons are as follows: ther was rapid onset of the problem after FMT. Furthermore, the patient never had ileus problems before.

To sum up, several medical phenomena are improbable concerning the causality, but this does not mean that it does not happen. Coincidence seems likely yet from our present understanding, but our understanding in the complex interactions between gut microbiota and the host, although rapidly evolving, is still sketchy. Yet, current evidence deems FMT as a generally safe therapeutic method with few adverse effects, but the long-term outcomes of FMT have not been completely elucidated. Furthermore, in an analysis of Wang et al. [7] the authors systematically reviewed 7562 original papers in the literature by July 2015 to evaluate the side effects of FMT. Finally, 534 studies with data from 1089 patients aged 1 to 95 years were included in the evaluation. The following adverse events have been reported: nonspecific ones such as fever, convulsions, diarrhea, nausea, flatulence, runny nose, headache, sore throat, and chills, but also severe ones such as bleeding, intestinal inflammation, severe diarrhea, severe intestinal spasms, and death. After division into possible, probable, and very likely assumed assignments to the FMT, there remained in spite of the large number of patients usually only low numbers, or single cases. Rather unlikely side effects in the context with FMT such as norovirus and cytomegalovirus infections, bacteremia, appendicitis, urethral infection, diverticulitis, influenza B, septic shock or lethal effects of peritonitis, and pneumonia or bronchiolitis were each reported in only one or two patients. However, these phenomena reported do also seem as “unlikely” as an ileus. This encouraged us to present our case. We would be very much interested, if comparable phenomena not easily to understand as simple coincidence were also observed by other investigators and hope for a lively discussion.
